# *Schisandra chinensis* essential oil attenuates acetaminophen-induced liver injury through alleviating oxidative stress and activating autophagy

**DOI:** 10.1080/13880209.2022.2067569

**Published:** 2022-05-19

**Authors:** Jing Zhao, Kaixin Ding, Manting Hou, Yuanhua Li, Xiaorong Hou, Wenzhang Dai, Zhiyong Li, Jun Zhao, Wenlong Liu, Zhaofang Bai

**Affiliations:** a School of Pharmacy, Hunan University of Chinese Medicine, Changsha, China; b Department of Hepatology, Fifth Medical Center of Chinese PLA General Hospital, Beijing, China; c China Military Institute of Chinese Materia, the Fifth Medical Centre, Chinese PLA General Hospital, Beijing, China

**Keywords:** Drug-induced liver injury, oxidative stress, essential oil, antioxidant, autophagy

## Abstract

**Context:**

*Schisandra chinensis* (Turcz.) Baill. (Magnoliaceae) essential oil (SCEO) composition is rich in lignans that are believed to perform protective effects in the liver.

**Objective:**

This study investigates the effects of SCEO in the treatment of acetaminophen (APAP)-induced liver injury in mice.

**Materials and methods:**

C57BL/6 mice (*n* = 56) were randomly divided into seven groups: normal; APAP (300 mg/kg); APAP plus bicyclol (200 mg/kg); APAP plus SCEO (0.25, 0.5, 1, 2 g/kg). Serum biochemical parameters for liver function, inflammatory factors, and antioxidant activities were determined. The protein expression levels of Nrf2, GCLC, GCLM, HO-1, p62, and LC3 were assessed by western blotting. Nrf2, GCLC, HO-1, p62, and LC3 mRNA were detected by real-time PCR.

**Results:**

Compared to APAP overdose, SCEO (2 g/kg) pre-treatment reduced the serum levels of AST (79.4%), ALT (84.6%), TNF-α (57.3%), and IL-6 (53.0%). In addition, SCEO (2 g/kg) markedly suppressed cytochrome P450 2E1 (CYP2E1) (15.4%) and attenuated the exhaustion of GSH (43.6%) and SOD (16.8%), and the accumulation of MDA (22.6%) in the liver, to inhibit the occurrence of oxidative stress. Moreover, hepatic tissues from our experiment revealed that SCEO pre-treatment mitigated liver injury caused by oxidative stress by increasing Nrf2, HO-1, and GCL. Additionally, SCEO activated autophagy, which upregulated hepatic LC3-II and decreased p62 in APAP overdose mice (*p* < 0.05).

**Discussion and conclusions:**

Our evidence demonstrated that SCEO protects hepatocytes from APAP-induced liver injury *in vivo* and the findings will provide a reliable theoretical basis for developing novel therapeutics.

## Introduction

As a worldwide antipyretic and analgesic drug, acetaminophen (APAP) is effective at therapeutic doses (Cao et al. 2018), but still has some side effects in causing Steven Johnson syndrome or other problems. When administered in an overdose, the result can be severe drug-induced liver damage, cell death, acute liver failure, and even high mortality in animals and humans (Wang et al. 2019). Within therapeutic doses, approximately 90% of APAP is metabolised to glucuronidation, and sulphation is eliminated in urine (Cai et al. 2018). The remaining small amounts are converted via cytochrome P450 enzymes (CYP2E1, CYP2A6) into *N*-acetyl-*p*-benzoquinone imine (NAPQI), which rapidly conjugates with cellular GSH and is excreted through bile or urine (Yan et al. 2016; Yang et al. 2019). Nevertheless, when an overdose occurs, both sulphation and glucuronidation pathways are saturated, and a large amount of NAPQI generation consumes GSH, resulting in oxidative stress, hepatic necrosis, and even high mortality (Wu et al. 2019). Although the mechanisms underlying APAP-induced liver injury (ALI) are extensively understood, novel drugs against the damage efficiency are necessary.

Liver injury is closely related to oxidative stress, inflammation, and autophagy (Iorga et al. 2017). Nrf2 plays a key role in maintaining cellular homeostasis and protects against the cytotoxicity of oxidative stress (Bellezza et al. 2018; He et al. 2020; Sun et al. 2020). When cells are exposed to oxidative stress, Nrf2 is detached from Kelch-like ECH-associated protein 1 (Keap1) and expresses its antioxidant enzymes, including NAD(P)H, quinone oxidoreductase 1 (NQO1), HO-1, and GCL by binding to antioxidant response elements to restore cellular homeostasis (Loboda et al. 2016; Xu et al. 2018; Chen et al. 2019). The activation of Nrf2 could not only alleviate the liver damage caused by oxidative stress but also have a protective effect on related inflammation. In addition, autophagy is a vital cellular process in maintaining cell homeostasis via the degradation of long-lived cytoplasmic proteins or dysfunctional organelles (Chao et al. 2018). Studies have demonstrated that cellular autophagy can be activated by APAP to remove damaged mitochondria and APAP protein adducts as a vital antioxidant mechanism to attenuate oxidative stress (Ni et al. 2016). P62 is an autophagy adaptor gene in selective autophagy, and an increase in autophagy flux is always accompanied by cell depletion of p62, which means that a decrease in p62 abundance is usually interpreted as a substrate protein of autophagy. P62 co-localizes the ubiquitinated proteins into the autophagic machinery to degrade them, including binding to the microtubule-associated protein 1 A/1B light chain 3B (LC3B) (Saito et al. 2020).

*Schisandra chinensis* (Turcz.) Baill. (Magnoliaceae) (SC) is a woody plant that is mainly located in Korea, Japan, and China (Xu et al. 2019). Among those countries, China has the most abundant resources of SC, and there is a total of 25 species of Schisandra plants worldwide, with 19 species distributed throughout China (Li et al. 2018). Therefore, the accessibility of SC results in its wide use in China, several SC teas, SC yogurt, SC wine, functional foods, health care products, and medicines have been produced using SC (Li et al. 2018). Many pharmacological studies have demonstrated that SC has various bioactivities, including antioxidant, hepatoprotective, anti-inflammatory, and neuroprotective effects (Panossian and Wikman 2008; Chun et al. 2014).

SC contains several active constituents such as lignans, essential oils, polysaccharides, and organic acids. Among these active constituents, essential oils and lignans have been supported as the main bioactive components in recent studies (Xu et al. 2019). Essential oils are complex natural mixtures of various secondary metabolites, mainly found in the leaves, bark, or fruits of aromatic plants (Bakkali et al. 2008). The chemical constitutions of SCEO have been identified, of which the majority of organic compounds are monoterpenes, sesquiterpenes, aromatic compounds, and lignans, and have performed various pharmacological capacities, including antioxidant, antimicrobial, and antiseptic effects. In addition, lignans have been proved to have a solid hepatoprotective effect (An et al. 2015; Jeong et al. 2015; Zhu et al. 2019). However, the underlying mechanisms by which SCEO protects against ALI have been poorly characterised.

Therefore, we aimed to evaluate the hepatoprotective effects of SCEO against ALI, to provide a theoretical basis for further development of new SCEO drugs for the prevention and treatment of drug-induced liver injury and healthy foods with auxiliary functions. Bicyclol, an anti-hepatitis drug developed by China, has hepatoprotective effects against experimental liver injury induced by diverse chemical stimulations. It was used as the positive control in our study because part of the liver protection mechanism of bicyclol was to eliminate oxidative stress (Wang et al. 2018).

## Materials and methods

### Chemicals and materials

Acetaminophen (HY-66005), SYBR Green qPCR Master Mix (HY-K0522), and RT Master Mix for qPCR (HY-K0510) were supplied by Medchem Express (Monmouth Junction, NJ, USA). Bicyclol tablets (H20051712) were purchased from the Beijing Union Pharmaceutical Factory (Beijing, China). SCEO was pooled from *SC* via direct pressing and obtained from Shandong Shibojindu Pharmaceutical Company (Shandong, China). Schisandrol A, schisandrol B, schizandrol A, schizandrol B, schizandrin A, schizandrin B, and schizandrin C were of HPLC grade and were obtained from Chengdu Pufei De Biotech Co., Ltd. (Chengdu, China). Quick Start Bradford 1x Dye Reagent (500-0205) was purchased from Bio-Rad Laboratories, Inc. (Hercules, CA, USA). TRIzol reagent was purchased from Invitrogen (Carlsbad, CA, USA). The primary antibodies anti-mouse Nrf2, HO-1, GCLC, GCLM, and β-actin were obtained from Abcam (Cambridge, UK), and p62 and LC3B were purchased from Cell Signalling Technology (Cambridge, UK). Commercial ALT (c009-2-1) and AST (c010-2-1) commercial kits were provided by the Nanjing Jiancheng Bioengineering Research Institute (Nanjing, China). Enzyme-linked immunosorbent assay (ELISA) kits for mouse GSH (KL-GSH-Mu), SOD (KL-SOD-Mu), CYP2E1 (KL-CYP450-Mu), and MDA (KL-MDA-Mu) were obtained from Shanghai Kang Lang Biological Technology Co., Ltd. (Shanghai, China). Mouse TNF-α (1217202) and IL-6 (1210602) ELISA kits were obtained from Dakewe Biotech Co., Ltd. (Shenzhen, China). All other reagents were of analytical grade and obtained from Sinopharm Chemical Reagent Co., Ltd. (Shanghai, China).

### Ultra-performance liquid chromatography (UPLC) analysis of SCEO

SCEO was qualitatively and quantitatively determined via UPLC. A sample of SCEO was mixed 10 μL into 5 mL methanol and filtered through a 0.22 μm filter, and then the sample was eluted in a UPLC system. The detection was analysed in a Waters UPLC system with chromatographic conditions as follows: column, ACQUITY BEH C18 (2.1 × 100 mm, 1.7 μm); mobile phase A and B: acetonitrile and 0.1% phosphoric acid aqueous solution; elution program (0–2 min 5% A → 55% A; 2–15 min, 55% A → 67% A; 15–20 min, 67% A → 78% A; 20–23 min, 78% A → 95% A; 23–30 min, 95% A → 5% A); flow rate: 0.2 mL/min; injection volume: 1 μL and Detection wavelength: 250 nm. On the basis of UPLC analysis, we identified several key compounds from the SCEO samples and matched their corresponding peaks.

### Experimental animals and protocol

**Figure 1.**
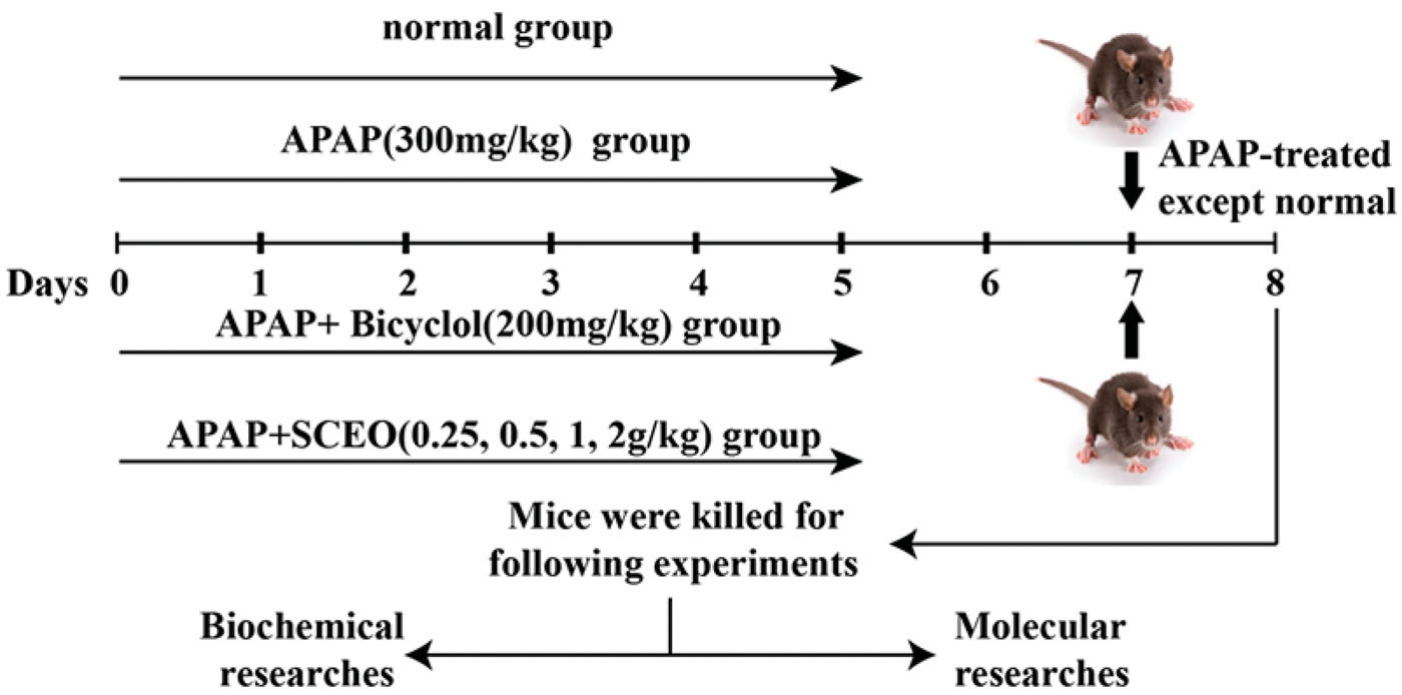
Experimental protocol for APAP-induced liver injury (ALI) model and SCEO treatment processes.

Adult male C57BL/6 mice (weight 20–22 g) were purchased from SPF Biotechnology Co., Ltd. (China). All animals were permitted free access to food and water and maintained under controlled cages (22 ± 2 °C, 12 h light/dark cycle). After adaptive bleeding for 1 week, animals were randomly divided into seven groups (*n* = 8): (1) normal; (2) APAP (300 mg/kg); (3) APAP plus bicyclol (200 mg/kg); (4) APAP plus SCEO (0.25 g/kg); (5) APAP plus SCEO (0.5 g/kg), (6) APAP plus SCEO (1 g/kg), and (7) APAP plus SCEO (2 g/kg). The protocol of the mouse experiment is presented in Figure 1.

SCEO was suspended in 0.5% (w/v) sodium carboxymethyl cellulose (CMC-Na) solution and gavaged into animals in the four experimental groups for seven consecutive days; the control and APAP groups were treated with 0.5% CMC-Na solutions. APAP was administered to mice after the final dose treatment, except for the control group, and all mice were euthanized 24 h after the APAP challenge, and samples were collected. The liver tissue was removed for detection, and the tissue was cleaned with normal saline and blotted dry, weighed, and recorded as, for example, shape and size. The liver lobes were fixed in 10% (w/v) formalin buffer for histopathological analysis, and the remaining liver tissues were stored at −80 °C for later use. All the animal experimental procedures in the study adhered to the guidelines for the care and use of laboratory animals at the Fifth Medical Centre of PLA General Hospital, Beijing, China (License No: IACUC-2021-0003). We exhausted our effort to reduce the number and pain of the mice used in this study.

### Analysis of serum ALT and AST levels

Blood was centrifuged at 3500 rpm for 15 min twice to obtain serum. The serum levels of ALT and AST were used in the kits.

### Analysis of hepatic GSH, SOD, MDA, and CYP2E1 levels

Briefly, the remaining liver tissue (30–50 mg) was prepared into homogenate samples and lysed in radio immunoprecipitation assay (RIPA) lysis buffer, followed by centrifugation (10000 rpm/min, 10 min); the protein concentration was determined by Bradford assay; and the supernatant of all samples was homogenised in a protein concentration by RIPA lysis buffer and prepared for subsequent detection. GSH, SOD, MDA, and CYP2E1 levels in hepatic tissue were measured using homogenised samples according to the commercial kit protocols.

### Analysis of serum TNF-α and IL-6 inflammation levels

The levels of inflammatory cytokines (TNF-α and IL-6) in the serum were evaluated using an ELISA system according to the manufacturer's instructions.

### Histopathological examination

With the study completed, each hepatic tissue was preserved in 10% buffered formaldehyde for 24 h and processed at room temperature before paraffin embedding. Embedding tissue was slipped into 5 μm slices with a microtome and mounted on glass slides. Finally, they were stained with haematoxylin and eosin (H&E) for morphological evaluation.

### Western blotting

Protein was collected from the liver tissue by RIPA lysis buffer that contained protease inhibitors, and the amount was homogenised via the Bradford method. Equal protein samples were separated by 10% or 12% Sodium dodecyl sulfate-polyacrylamide gelelectrophoresis (SDS-PAGE) and transferred to polyvinylidene fluoride (PVDF). Afterward, the membrane was blocked with 5% skim milk for 1 h at room temperature, probed with primary antibodies overnight at 4 °C, and incubated with HRP-conjugated secondary antibody (1:5000) for 1 h at room temperature. The signals were captured with the enhanced chemiluminescent reagents (Promega, Beijing, China) detection system and measured using ImageJ software.

### RNA extraction and real-time polymerase chain reaction (real-time PCR)

Hepatic tissue was homogenised and TRIzol reagent was used to extract total RNA, and RT Master Mix for PCR kit was used to reverse transcription based on the kit’s protocol. Real-time PCR was performed using the SYBR Green qPCR Master Mix kit and normalised to β-actin. The specific primer sequences designed and used for real-time PCR are provided in Table 1.

**Table 1.** Primer sequences used for real-time PCR detection.

Gene	Type	Sequence (5′-3′)
P62	Forward	CGTTTGACGGAAGGTAAAT
	Reverse	TCATCAGCGGGCTGTATC
LC3	Forward	GATAATCAGACGGCGCTTGC
	Reverse	ACTTCGGAGATGGGAGTGGA
TNF-α	Forward	CCACCACGCTCTTCTGTCTAC
	Reverse	GAGGGTCTGGGCCATAGAA
IL-6	Forward	ACAACCACGGCCTTCCCTACTT
	Reverse	CACGATTTCCCAGAGAACATGTG
CYP2E1	Forward	CGTTGCCTTGCTTGTCTGGA
	Reverse	AAGAAAGGAATTGGGAAAGGTCC
HO-1	Forward	TGCAGGTGATGCTGACAGAGG
	Reverse	GGGATGAGCTAGTGCTGATCTGG
NRF2	Forward	CGAGATATACGCAGGAGAGGTAAGA
	Reverse	GCTCGACAATGTTCTCCAGCTT
GCLC	Forward	CAGTCAAGGACCGGCACAAG
	Reverse	CAAGAACATCGCCTCCATTCAG
β-actin	Forward	GGCTGTATTCCCCTCCATCG
	Reverse	CCAGTTGGTAACAATGCCATGT

### Statistical analysis

Data were expressed as mean value ± SD. GraphPad Prism eight software was used for the statistical analysis. The experiment was independently repeated three times. Unpaired *t*-tests were used for the mean values. Statistical significance was set at *p* < 0.05.

## Results

### UPLC analysis and composition of SCEO

**Figure 2.**
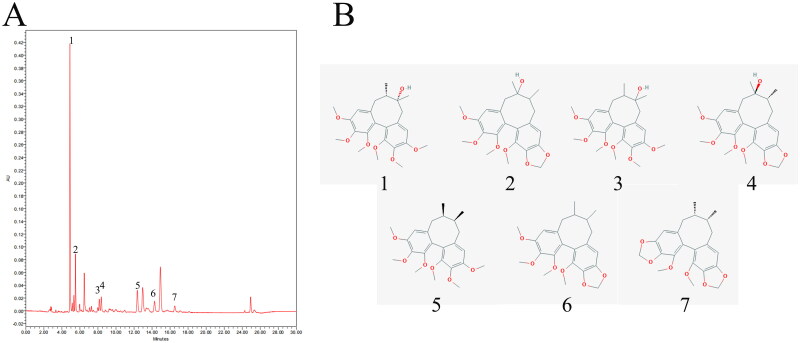
Representative UPLC fingerprint of SCEO. (A) The chromatographic profile of SCEO analysed by UPLC; (B) Chemical structure of the main constituents of SCEO. (1) schisandrol A (2) schisandrol B (3) schizandrol A (4) schizandrol B (5) schizandrin A (6) schizandrin B, and (7) schizandrin C.

The chromatographic profiles of the components of the SCEO were analysed using UPLC (Figure 2A). UPLC analysis showed the following elution times: schisandrol A (5.06 min), schisandrol B (5.66 min), schizandrol A (8.362 min), schizandrol B (8.58 min), schizandrin A (12.95 min), schizandrin B (14.88 min), and schizandrin C (17.24 min). The data in Figure 2B show the chemical structures of chosen seven index components of SCEO. The concentrations of the seven index compounds with three different batches of SCEO samples were detected, and the results are listed in Table 2. The mean mass fractions of schisandrol A, schisandrol B, schizandrol A, schizandrol B, schizandrin A, schizandrin B, and schizandrin C were 17.46, 5.06, 1.19, 1.96, 3.68, 5.76, and 0.69 mg/g, respectively.

**Table 2.** Concentrations of 7 index components of SCEO.

Lot number	Mass fraction (mg/g)						
	Schisandrol A	Schisandrol B	Schizandrol A	Schizandrol B	Schizandrin A	Schizandrin B	Schizandrin C
20200407	18.52	5.04	1.29	2.04	4.08	5.92	0.72
200411	17.13	5.23	1.11	1.87	3.32	5.93	0.81
2020411	16.72	4.92	1.18	1.96	3.64	5.42	0.54
Mean	17.46	5.06	1.19	1.96	3.68	5.76	0.69

### SCEO alleviated APAP-induced hepatic injury

**Figure 3.**
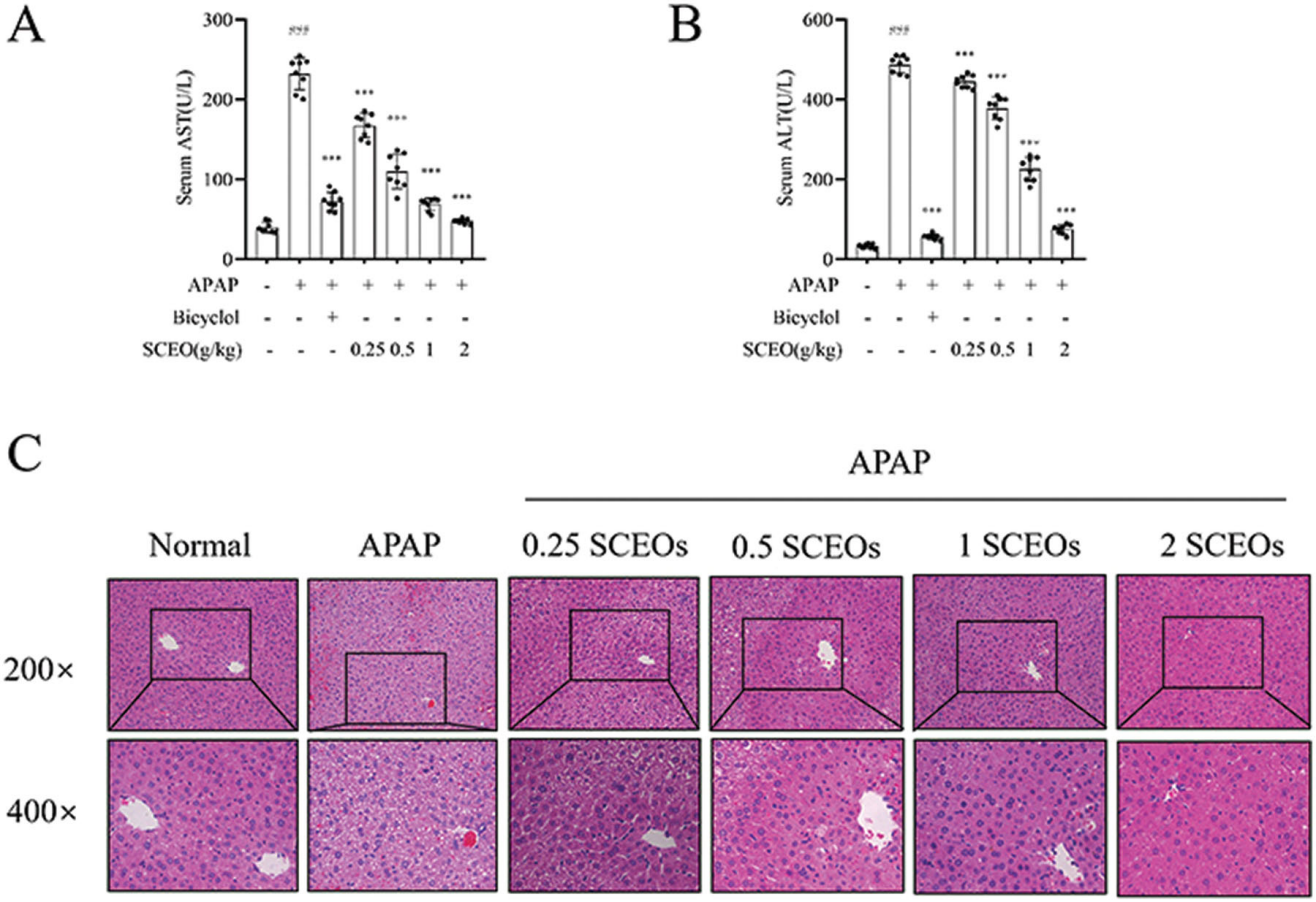
Effect of SCEO on alleviating APAP-induced liver injury. (A,B) Serum was collected for assessment of AST and ALT activity. (C) Representative histological images of the liver section with H&E stain (200× and 400× magnification). Data are expressed as mean ± SD (*n* = 8). ***p < 0.001 compared to the APAP group. ###*p* < 0.001 compared to the normal group.

To investigate the effect of SCEO in alleviating APAP-induced hepatic injury, we treated the mice with SCEO (0.25, 0.5, 1, and 2 g/kg) before administration of 300 mg/kg APAP. ALT and AST, two serum biochemical indicators of liver injury, were used to assess the effects of SCEO. Compared with the normal group, levels of ALT and AST in all mice increased with APAP overdose (Figures 3A,B). Furthermore, with the treatment of SCEO (0.25, 0.5, 1, and 2 g/kg) and bicyclol (200 mg/kg) significantly reduced serum ALT and AST levels. Furthermore, we performed H&E staining of the hepatic tissue to assess liver histological integrity and architecture. As shown in Figure 3C, normal hepatic cells were arranged neatly around blood vessels. However, the overdose of APAP-induced severe liver injury resulted in massive hepatocyte necrosis, cytoplasmic vacuolisation, and lymphocyte infiltration. Pre-treatment with SCEO ameliorated hepatocyte congestion and centrilobular necrosis in mice liver tissues after APAP administration.

Even at the lowest dosage of 0.25 g/kg, the amelioration of ALI by SCEO was significant, and the APAP groups pre-treated with 1 and 2 g/kg SCEO were unscathed and appeared almost normal. Histological analysis showed that the SCEO pre-treatment resulted in a protective effect against APAP-induced hepatotoxicity. These results indicated that SCEO produced a significant protective effect against APAP-induced hepatotoxicity.

### Effects of SCEO on suppressing APAP-induced proinflammatory cytokines

Inflammation is a critical cause of APAP-induced hepatic injury. Proinflammatory cytokines are mediators of inflammation, and several proinflammatory cytokines, such as TNF-α and IL-6, increase the innate immune response and cause severe liver injury after APAP overdose. Real-time PCR and ELISA kits were used to detect the inhibitory effects of SCEO on liver mRNA expression and serum levels of proinflammatory cytokines induced by APAP overdose.

**Figure 4.**
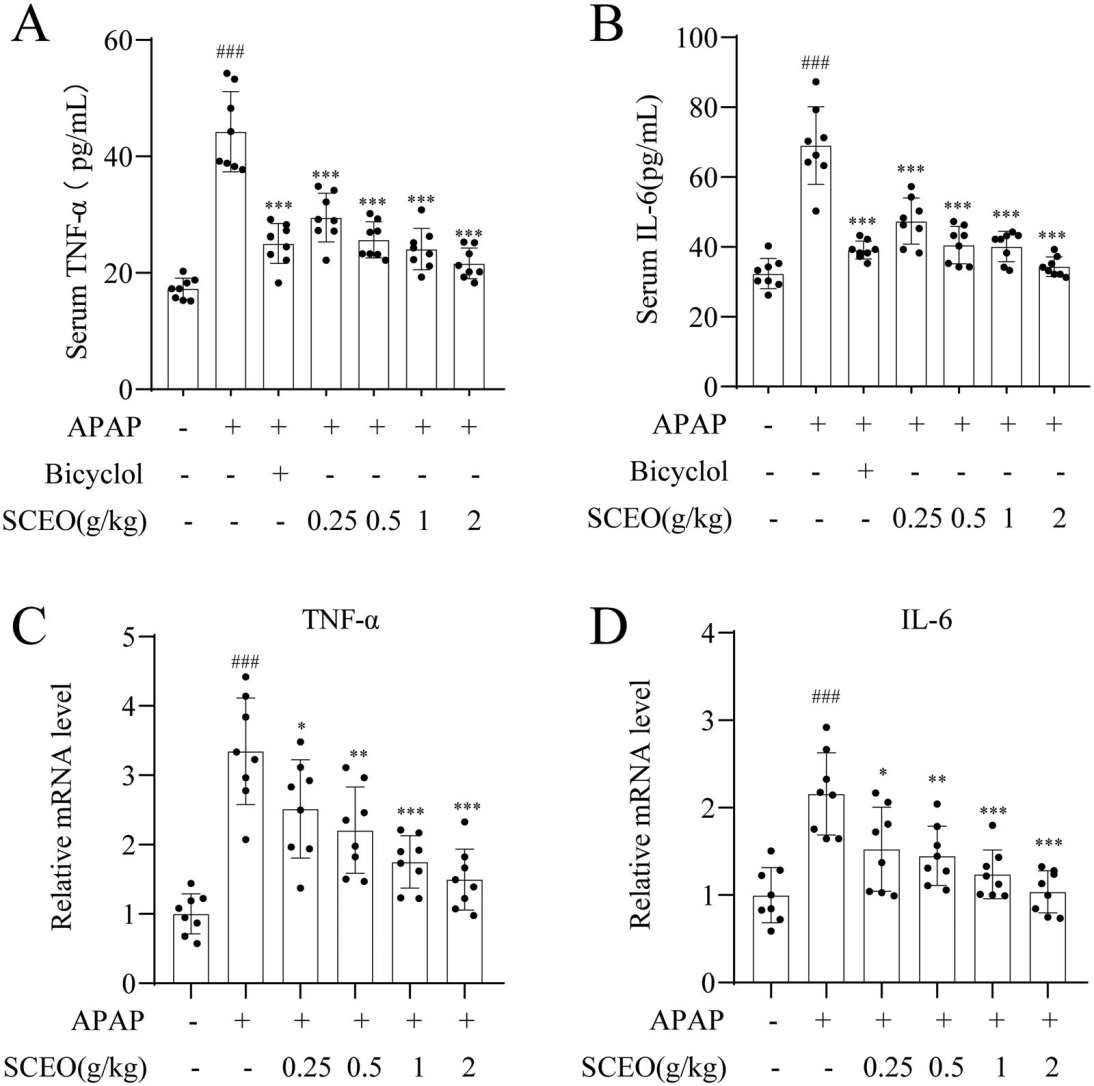
Pre-treatment with SCEO in preventing APAP-induced liver failure. The levels of (A) TNF-α, and (B) IL-6 in the serum were measured by ELISA kits, the hepatic mRNA expression of (C) TNF-α, and (D) IL-6 were determined by real-time PCR. Data were expressed as mean ± S.D (*n* = 8). ***P < 0.001 compared with the APAP group and ###*P* < 0.001 compared with control group.

As indicated in Figure 4, the TNF-α and IL-6 levels were obviously increased in overdose APAP in mice; however, the release of the cytokines was suppressed by SCEO pre-treatment. In addition, compared with the normal group, the overdose of APAP significantly increased the mRNA expression of TNF-α and IL-6, corresponding to the SCEO pre-treatment markedly reduced these increases. These data suggest that the reduction of inflammatory cytokines in mice is related to the hepatoprotective effect of SCEO on APAP-induced ALI.

### Effects of SCEO pre-treatment reduced oxidative stress in APAP-induced mice

Oxidative stress plays a vital role in ALI. However, APAP does not induce hepatotoxicity. CYP2E1, a metabolic enzyme, can metabolise excessive intake of APAP into the NAPQI. Furthermore, excessive NAPQI exhausted hepatic GSH, resulting in oxidative injury, which is an understood mechanism for APAP-induced hepatocellular necrosis. To explore the antioxidation of SCEO in APAP-induced liver failure, hepatic oxidative stress indices, such as GSH, SOD, MDA, and CYP2E1, were determined using ELISA kits. The change in MDA content can reflect the oxidative damage to cell membrane lipids.

**Figure 5.**
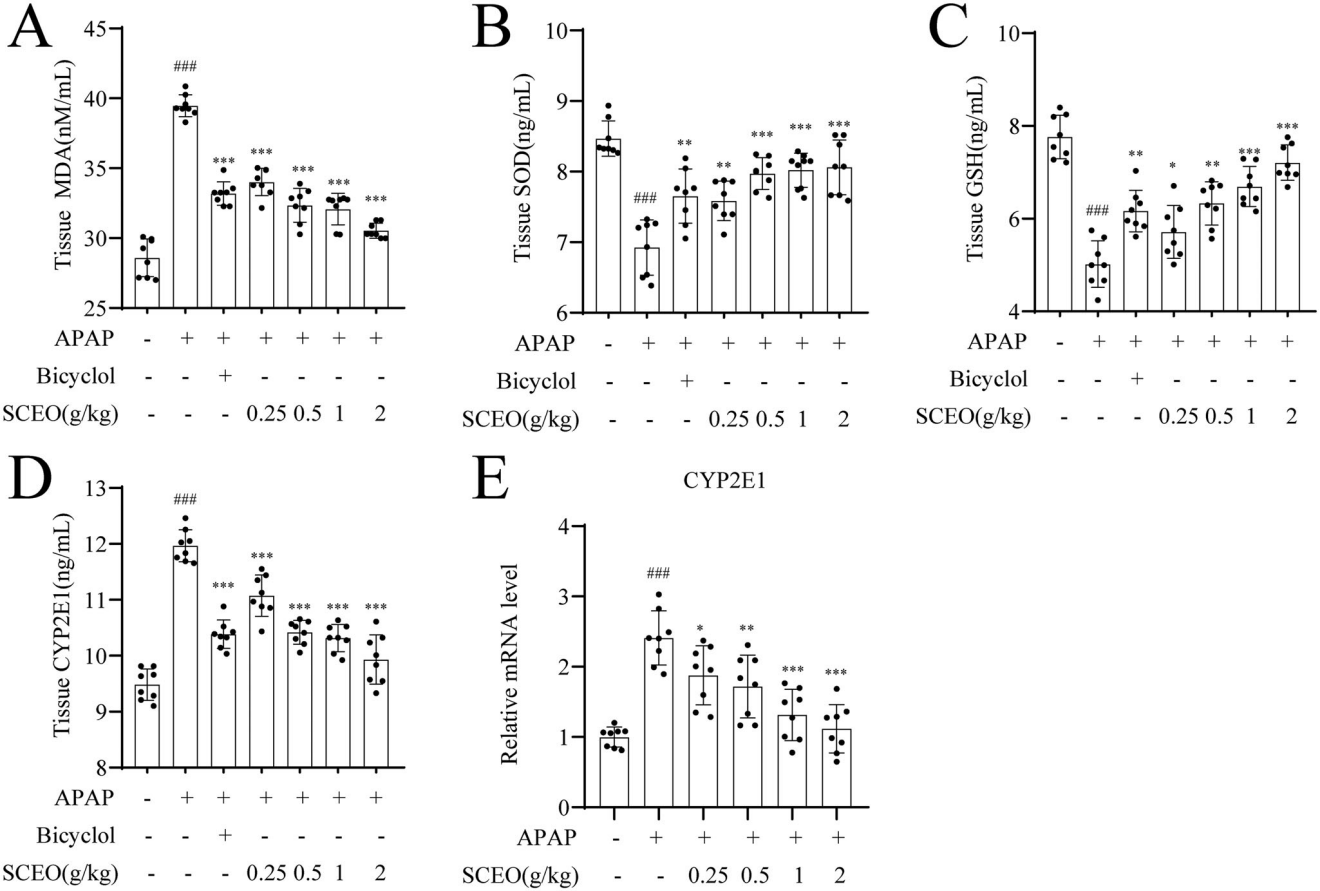
SCEO alleviated hepatic oxidative stress in APAP-induced hepatotoxicity mice. (A) Tissue MDA levels. (B) Tissue SOD activities. (C) Tissue GSH activities. (D) Tissue CYP2E1 levels. (E) mRNA expression levels of CYP2E1. Data are expressed as mean ± SD (*n* = 8). ###*p* <  0.001 compared to the normal group; ***p < 0.001 compared to the APAP group.

As shown in Figure 5A, an overdose of APAP enhanced hepatic MDA levels. However, the increase in MDA was suppressed by pre-treatment with SCEO in a dose-dependent manner. Similarly, hepatic GSH and SOD are the main regulators responsible for the redox homeostasis of tissues in response to oxidative stress. As expected, GSH and SOD levels in the liver tissue were significantly alleviated by pre-treatment with SCEO. Furthermore, APAP overdose markedly enhanced CYP2E1 production, and pre-treatment with SCEO effectively disputed its alterations. The CYP2E1 expression trend was confirmed at the mRNA level, suggesting SCEO pre-treatment attenuates CYP2E1 to suppress APAP-induced liver failure. The aforementioned results demonstrated that SCEO protected the liver from APAP-induced damage via repression of oxidative stress.

### Protective effect of SCEO against APAP-induced liver damage was dependent on the Nrf2 pathway

To investigate the possible antioxidant mechanisms of SCEO pre-treatment against oxidative stress, we assessed the classical Nrf2 signalling pathway, a notable antioxidant response signalling pathway (Yang et al. 2013). The mRNA and protein expression levels of Nrf2, and its downstream target genes, GCLC, GCLM, and HO-1, were determined by real-time PCR and western blotting.

**Figure 6.**
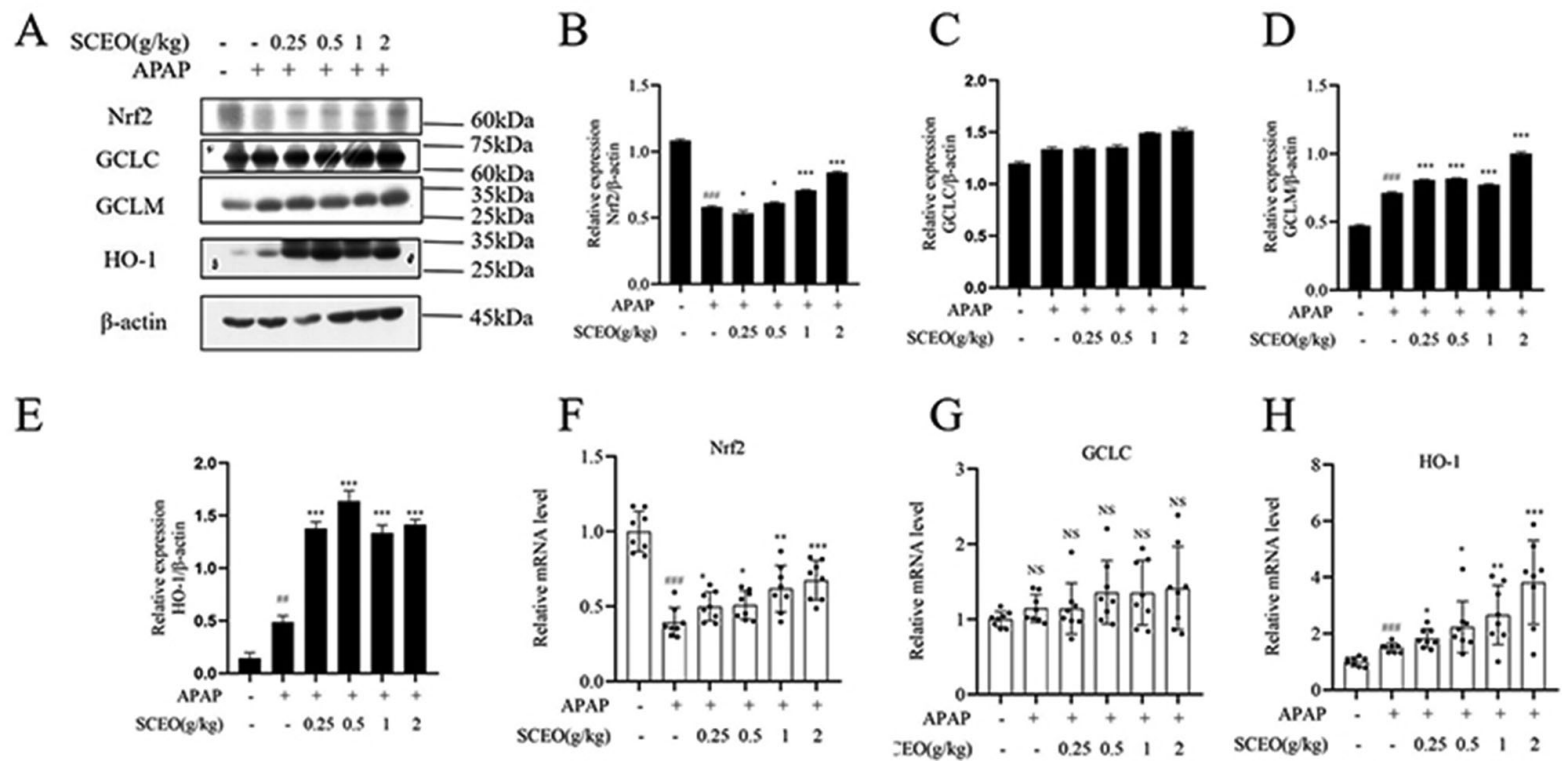
The effect of SCEO on the Nrf2 signal pathway. (A–E) protein expression of Nrf2, HO-1, GCLC, GCLM, (F–G) mRNA level of Nrf2, GCLC, and HO-1 in liver tissues of mice exposed to APAP overdose, β- actin served as a loading control. The values are reported as the means ± SD (*n* = 8). ###*p* < 0.001 compared with the normal group; *p < 0.05, **p < 0.01, ***p < 0.001 compared with the APAP group.

As shown in Figure 6, the Nrf2 protein level in mice with APAP overdose was significantly reduced with the normal group, and SCEO supplementation restored Nrf2 protein levels in the liver tissue. Furthermore, the expression of downstream target genes HO-1, GCLC, and GCLM were detected. APAP had a significant effect on GCLM and HO-1. Moreover, SCEO pre-treatment enhanced the protein expression of GCLM and HO-1. However, there was no obvious difference in the protein expression of GCLC with or without APAP, and SCEO also had no significant effect on GCLC protein expression. Next, APAP overdose decreased the mRNA expression of Nrf2, and the decreased mRNA expression of Nrf2 induced by APAP was reversed by SCEO pre-treatment. The results in Figure 6 also demonstrate that APAP and SCEO had no significant effect on the mRNA expression of GCLC. Furthermore, SCEO pre-treatment enhanced the mRNA expression of both GCLM and HO-1. These data indicate that SCEO enhances the antioxidant defense system to prevent ALI.

### SCEO activates autophagy in response to APAP-induced liver damage

Although the harmful mechanisms induced by APAP overdose have been well explored, little is known of the cellular adaptive mechanisms that may attenuate ALI. Cells protect themselves and ensure their survival by removing damaged mitochondria, called autophagy (Ni et al. 2013). Therefore, we further investigated whether SCEO could regulate autophagy to protect against ALI.

**Figure 7.**
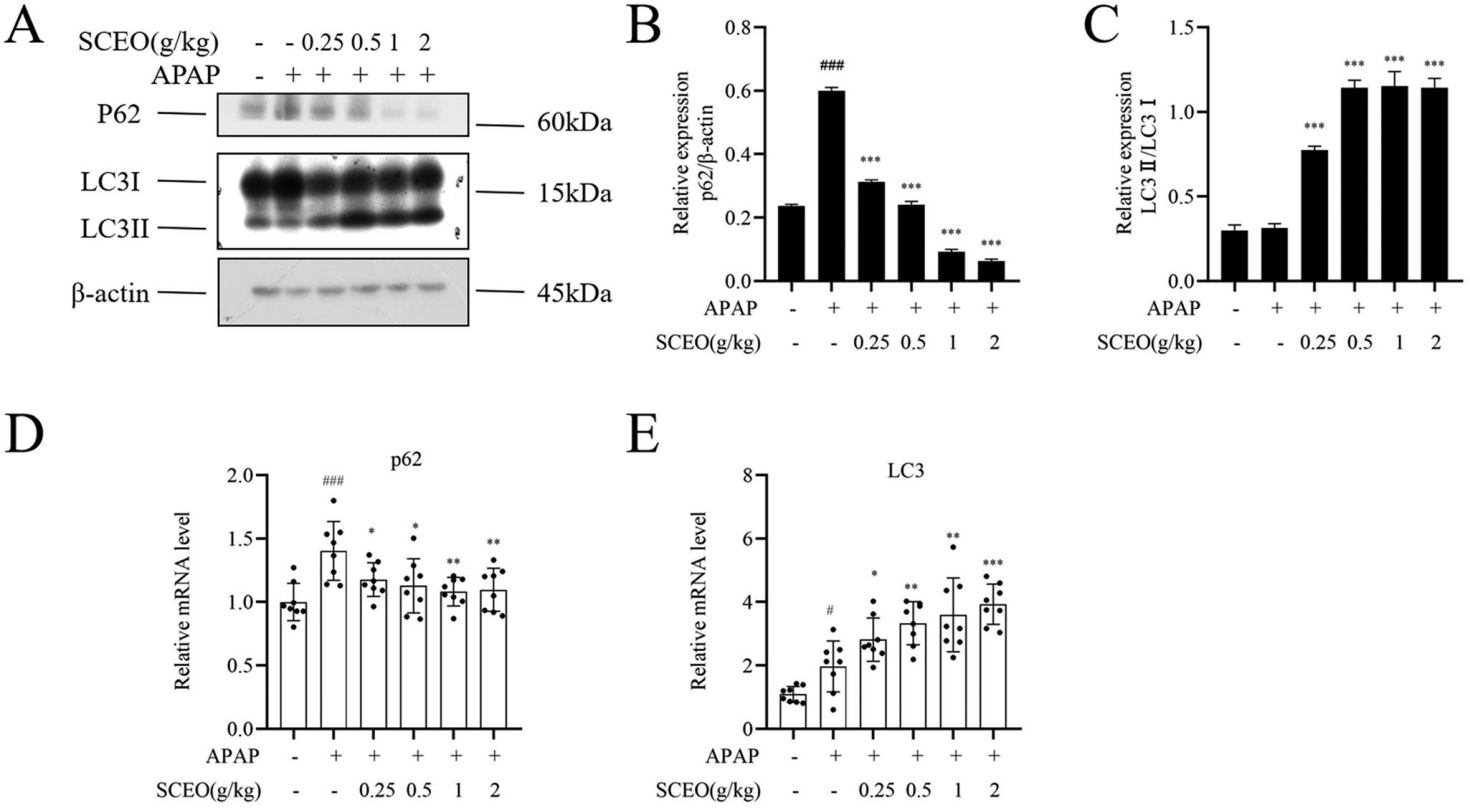
SCEO upregulated hepatic autophagy in APAP-induced hepatotoxicity mice. (A–C) The protein level of p62, LC3 I, and LC3 II were determined (D–E) mRNA levels of p62, and LC3, β-actin served as a loading control. The values are reported as the means ± SD (*n* = 8). ###*p* < 0.001 compared with the normal group; *p < 0.05, **p < 0.01, ***p < 0.001 compared with the APAP group.

As shown in Figure 7, after APAP overdose, the protein expression level of LC3-II, a reliable marker of autophagosomes, did not change compared with that of the normal group. However, SCEO pre-treatment significantly enhanced LC3-II protein expression. The protein expression level of p62, a selective substrate for autophagy, significantly increased compared with that in the normal group after APAP overdose, and this enhancement was attenuated via SCEO. In addition, the mRNA levels of LC3 increased after SCEO pre-treatment to protect the liver.

## Discussion

APAP is used worldwide to alleviate pain and fever as an accessible over-the-counter (OTC) drug (Dkhil et al. 2019). In general, APAP has few side effects with therapeutic doses. However, overdose can cause acute liver injury as the most common cause of ALI, resulting in various complications, including inflammation and oxidative stress (Bunchorntavakul and Reddy 2018; McCrae et al. 2018). In this study, we established an acute liver injury model via injection of APAP overdose (300 mg/kg), which is evidence of an increase in serum liver function indicators and histological changes in hepatic tissue. AST and ALT, as two significant markers of liver function in serum, played a monitored role in assessing liver injury, and an increase in these indicators characterised a varying degree of hepatotoxicity, hepatocyte necrosis, or enhancement of permeability of their membranes (Ozer et al. 2008). Injection of APAP produced a significant increase in the activities of AST and ALT, and SCEO pre-treatment significantly inhibited the levels of ALT and AST, which were enhanced by APAP. Our results are supported by histopathological observations that showed obvious circumferential pericentral hepatitis, cytoplasmic vacuolisation, and lymphocyte infiltration, whereas SCEO pre-treatment produced a beneficial protective effect in alleviating ALI. The hepatoprotective effect of SCEO demonstrated that SCEO can maintain hepatic homeostasis and inhibit the release of liver enzymes.

SC has long been used as an herbal supplement in Chinese medicine and has been used in Western phytotherapy (Szopa et al. 2017; Kopustinskiene and Bernatoniene 2021). SC is a traditional Chinese medicine remedy for hepatoprotective, immunostimulant, antioxidant, ergogenic, and anti-stress (Panossian and Wikman 2008). SC contains approximately 1.5% sugars, 3% essential oils, various lignans, and other ingredients (Kopustinskiene and Bernatoniene 2021). The literature has demonstrated that lignans are key chemical compounds that are responsible for biological activity in SC. Furthermore, the most representative groups of SC lignans are dibenzocyclooctadiene lignans, which occur predominantly in SC are schisandrol A, schisandrol B, schizandrol A, schizandrol B, schizandrin A, schizandrin B, and schizandrin C (Szopa et al. 2017). According to our review of the literature, SC lignans have poor water solubility and are lipophilic. SCEO, extracted from SC, contained rich SC lignans, per our UPLC analysis, that can inhibit the release of transaminase, promote the recovery of damaged liver cells, increase the activity of deoxyribonucleic acid, and regenerate liver cells. SCEO has excellent liver detoxification function and is effective in anti-inflammation and antioxidant stress against various diseases (Jeong et al. 2015; McCrae et al. 2018). In this study, treatment with SCEO significantly relieved APAP-induced liver damage and suppressed the increase in serum aminotransferase and hepatic histopathological lesions, promoted autophagy flux, and alleviated oxidative stress in mice, suggesting that SCEO effectively attenuates APAP-induced hepatotoxicity.

Inflammatory response reflected by the liver toxicity of APAP and excessive activation of proinflammatory chemokines and cytokines were considered signs of liver injury. APAP-induced hepatotoxicity leads to the recruitment of neutrophils and monocytes in the injured area of the liver, produces more proinflammatory chemokines and cytokines and aggravated inflammatory response (Yang et al. 2017; Zhang et al. 2017). Inflammation is a double-edged sword: on the one hand, the inflammatory response could remove dead cells and debris that contribute to liver repair, and on the other hand, an over-inflammatory response can result in severe liver injury or hepatic death (Suzuki et al. 2016; Yang et al. 2017). Whether APAP-induced liver inflammation response aggravates liver injury remains controversial, and most studies have identified anti-inflammatory agents as a therapeutic strategy to protect against ALI (Imaeda et al. 2009). Our results indicate that SCEO pre-treatment significantly reversed the increase in the levels of TNF-α and IL-6 in the liver of APAP-overdose mice by using ELISA kits and real-time PCR detection.

The disequilibrium between oxidative stress and the antioxidant defense system is a major cause of ALI. APAP does not produce hepatotoxicity but generates excessive NAPQI metabolising by CYP2E1, which leads to oxidative stress. Oxidative stress can be scavenged by antioxidant systems, including low-molecular-weight antioxidants (e.g., GSH) and enzymes (e.g., SOD and MDA) (Wang et al. 2017; Ramachandran and Jaeschke 2019). In our study, the expression of CYP2E1 decreased with SCEO pre-treatment when an overdose of APAP (300 mg/kg) was administered. Furthermore, the expression of GSH, SOD, and MDA in hepatic tissues after SCEO pre-treatment was significantly different from that after APAP overdose. The results indicated that SCEO pre-treatment inhibited the generation of oxidative stress to some extent. Other studies have suggested that the activation of Nrf2 corresponds with the antioxidant properties of SCEO in ALI (Galicia-Moreno et al. 2020). The Nrf2 pathway is regarded as a vital cellular defense mechanism that regulates the expression of phase II detoxifying and antioxidant enzymes in preventing ALI. Under normal conditions, Nrf2 binds to a negative regulator Kelch-like ECH-associated protein (Keap1); under oxidative stress stimulation, Nrf2 dissociates from Keap1, transfers to the nucleus and binds to ARE, and upregulates the expression of downstream genes involved in antioxidant and detoxification, including GCLC, GCLM, NQO1, and HO-1 to alleviate oxidative stress (Lu et al. 2016; Silva-Islas and Maldonado 2018; Yamamoto et al. 2018; Baird and Yamamoto 2020). Our study showed that hepatic Nrf2 protein expression decreased in mice with APAP overdose; however, SCEO pre-treatment significantly increased the expression of both hepatic Nrf2 protein and typically recognised Nrf2 downstream genes, GCLC, HO-1, and GCLM. And SCEO pre-treatment decreased the expression of CYP 2E1 to regulate the levels of GSH, SOD, and MDA to protect against APAP toxicity in mice, both in inhibiting the occurrence of oxidative stress and defending against oxidative stress induced by APAP overdose.

Autophagy is generally considered a cytoprotective mechanism that resists various liver diseases through multiple functions, including degradation of long-lived cytosolic and damaged proteins, removal of damaged mitochondria, and regulation of cell death (Mukhopadhyay et al. 2018; Brecklinghaus 2020). Therefore, because mitochondrial damage and ATP depletion are key factors in APAP-induced hepatocyte necrosis, a reasonable assumption is that autophagy may be a critical hepatoprotective mechanism against ALI. In this study, APAP overdose increased autophagic flux at the protein levels and mRNA levels in cultured hepatocytes, as demonstrated by the autophagy substrate protein p62; however, the expression of LC3-II did not increase or decrease in protein level but appeared to increase in mRNA level. Notably, we found that autophagy activity in the SCEO group was enhanced, demonstrated by the increased expression of LC3-II and decreased expression of p62. Therefore, this enhancement of autophagy recognised SCEO protection against liver injury caused by APAP.

## Conclusions

Our results indicate that SCEO exerts its protective effect on APAP-induced hepatotoxicity by inhibiting the occurrence of oxidative stress, upregulating antioxidant signal pathway target gene expression, and activating autophagy. Therefore, we propose that SCEO could provide a reliable theoretical basis for developing novel therapeutics for drug-induced acute liver injury.
